# Energy and Protein Requirements for the Maintenance of Growing Male Sika Deer (*Cervus nippon*)

**DOI:** 10.3389/fvets.2021.745426

**Published:** 2021-09-14

**Authors:** Kun Bao, Xiaoxu Wang, Kaiying Wang, Guangyu Li, Hanlu Liu

**Affiliations:** Institute of Special Animal and Plant Sciences, Chinese Academy of Agricultural Sciences, Changchun, China

**Keywords:** carbon and nitrogen balance, maintenance requirement, methane emission, net energy, net protein

## Abstract

The objective of this study is to study the effects of dietary intake levels on energy metabolism, carbon (C), and nitrogen (N) balance and to determine the maintenance requirements of energy and protein for male sika deer during their growing period. A total of 16 1-year-old male sika deer with similar body weight (BW) (63.25 ± 2.42 kg) were selected, with four animals per feed intake level. The feeding levels of the four groups for deer were 40, 60, 80, and 100% of the recommended amount, respectively. The nutrient digestibility and methane production were measured through digestion trials and respiratory trials. A 4 × 4 Latin Square design was performed in a respirometry trial. The results show that the apparent digestibility of C and N gradually increased as the level of feed intake decreased. Furthermore, with a decrease in feed intake level, the metabolic energy intake (MEI), heat production (HP), and retained energy (RE) of male sika deer significantly decreased (*P* < 0.01). The requirements of metabolic energy for maintenance (MEm) and net energy for maintenance (NEm) of growing deer are 251.17 and 223.62 kJ kg^−1^BW^0.75^d^−1^, respectively, as estimated according to the logarithmic regression equations between HP and MEI. The net N requirement for maintenance (NNm) and net protein requirement for maintenance (NPm) of growing male sika deer based on the linear relationship between retained nitrogen (RN) and daily nitrogen intake (NI) were 251.8 mg kg^−1^BW^0.75^d^−1^ and 1.57 g kg^−1^BW^0.75^d^−1^, respectively. The NEm and NPm values obtained from this experiment fill the gap in net energy and protein requirements and serve as basic data for establishing the nutritional standards forsika deer breeding in China.

## Introduction

Sika deer (*Cervus nippon*) produce traditional Chinese medicine velvet antler and thus they are important ruminants in China. The nutritionals level of domestic sika deer are mainly drawn from foreign nutritional standards, such as NRC (1). However, since China has a vast territory, rich pasture resources, and many deer species, it is actually unreasonable to deal with all situations using a foreign standard. Moreover, the nutritional requirements for male sika deer during their growing period have not been fully determined in China, which limits the efficient development of sika deer industry. Therefore, it is essential to study the nutritional requirements of sika deer to improve production performance and ensure efficient utilization of feed.

To study the energy and protein maintenance requirements of 1-year-old male sika deer, carbon-nitrogen (C-N) balance method was used in this study, as well as the measurement of methane emissions through an open-circuit respiration measurement system. The C-N balance method has been used to calculate retained energy (RE), assuming that all energy is retained in the form of fat or protein (2). Therefore, this study further explores the effects of feeding levels on energy metabolism, C-N balance, and methane emission and uses the C-N balance method to determine the maintenance energy and protein requirements of male sika deer during the growing period.

## Materials and Methods

The study was carried out in the antler deer breeding base of the Institute of Special Animal and Plant Science, CAAS. All experiments were performed in accordance with the Animal Care and Use Guidelines of the Institute of Special Animal and Plant Science (Jilin, China).

### Animals and Treatments

A total of 16 1-year-old male sika deer with similar body weight (BW; 63.25 ± 2.42 kg) were selected, with four animals per intake level. The deer were divided into four treatment groups, and the experiment was carried in four experimental stages. Each stage lasted for 12 days. The feeding levels of the four groups for deer were 40, 60, 80, and 100% of the recommended amount, respectively, following the nutrition requirements of feed for deer (1). To reduce the inaccuracy of the test data caused by the deer' body conditions, the deer were given a rest for 5 days and fed a normal nutrition level diet after each stage. The deer were fed two equal meals at 06:30 and 15:30 daily, and they can drink freely. The dietary composition and nutritional content of basal diets are shown in Table 1.

**Table 1:** Composition and nutritive levels of control diet.

**Parameter**	**Concentration**
**Composition (%)**	
Corn flour	22
Soybean meal	12
Lucerne	50
Distillers dried grains with soluble (DDGS)	4
Corn germ meal	5.5
Molasses	5
NaCl	0.5
Conjugated linoleic acid	0.5
Additives^*^	0.5
Total	100
**Measured nutrient concentration(dry matter)**	
Gross energy (GE,MJ/kg)	14.03
Crude protein (CP, %)	15.80
Neutral detergent fiber (NDF, %)	41.54
Ether extract (EE, %)	3.31
Acid detergent fiber (ADF, %)	16.16
Ca (%)	0.76
P (%)	0.50

* *Contained the following per kg of premix: Mg, 76 mg; Cu, 36 mg; Zn, 43 mg; Fe, 53 mg; vitamin A,2484 IU; vitamin D_3_,496.8 IU; vitamin K_3_, 0.23 mg; vitamin B_1_,10.092 mg; vitamin B_2_,0.69 mg; vitamin B_12_,1.38 mg; folic acid, 0.023 mg; nicotinic acid, 1.62 mg; calcium pantothenate, 1.15 mg; CaHPO_4_,5.17 g; CaCO_3_,4.57 g*.

### Digestibility Trials

The digestibility of nutrients was measured by digestion trials. Deer were weighed at the beginning and end of the collection period. The feed refusals and feces were collected and weighed every day. Feces were collected every day for 3 days, stored at −20°C, then mixed and sampled again before chemical analysis, and dried at 65°C. Urine was collected in a bucket containing 20 mL of 10% concentrated sulfuric acid to avoid loss of nitrogen from urine. All the collected urine was weighed, and 3% of the daily urine output was sampled for analysis.

### Gas Metabolism

Four open-circuit respiration calorimetry chambers with a volume of 17.82 m^3^ (450 × 180 × 220 cm) were used in this study. In short, air conditioners and heaters were used to regulate the respiration chambers to maintain a constant temperature and humidity. A vacuum pump was used to continuously extract gas from the respiration chambers. The gas concentration in each respiration chamber was measured using an analyzer at a 3-min interval. O_2_ was measured with a zirconia sensor, while CO_2_ and CH_4_ were measured with a non-dispersive infrared sensor in the analyzer.

The concentration of CH_4_, CO_2_, and O_2_ was measured according to the method proposed by Tovar-Luna (3). Air was first analyzed for CH_4_, followed by CO_2_ and O_2_. Before each test, analyzers were calibrated with standard gas mixtures (19.5% and 20.5% O_2_, 0.0% and 1.5% CO_2_, and 0.0% and 0.3% CH_4_). The temperature and humidity in the calorimetry room were maintained at 20–23°C using an air conditioner at 50–55% using a dehumidifier, respectively (Whirlpool, Benton Harbor, MI).

An open-circuit respiratory heat measurement system was utilized at Deer Breeding Base of the Institute of Special Animal and Plant Science, CAAS (Jilin, China). A 4 × 4 Latin Square design was performed. Four deer were selected, with one deer put into one metabolism bin. After the 24-h adaptation period, CH_4_ and CO_2_ production of the individuals was measured for 24 consecutive hours. To avoid stress response in the deer in the metabolic cage, all animals had been trained previously. To reduce the inaccuracy of the test data caused by the deer' body conditions, the deer were given a rest for 5 days and fed a normal nutrition level diet after each stage.

### Chemical Analyses

The content of dry matter (DM), ash, Ca, and P in the feed, orts, and feces were analyzed by the method of AOAC (4). The concentrations of neutral detergent fiber (NDF) and acid detergent fiber (ADF) were determined according to the method described by Van Soest et al. (5). A bomb calorimeter (IKA C200, Germany) was used to measure gross energy (GE) in diets and feces. The GE of urine samples was measured with the method described by Deng et al. (6, 7). Carbon and nitrogen content in the feed, orts, feces, and urine was estimated in a C-N analyzer (Elementary Vario MAX CN, Germany).

### Data Calculation

#### Metabolizable Energy

The content of metabolizable energy (ME) in the diet was calculated based on the data obtained from the digestion trials. The difference between GE intake and fecal energy was thought to be digestible energy (DE). The ME of the diet with four feeding levels was obtained by subtracting CH_4_ energy and urinary energy from DE. Energy equivalent of CH_4_ was 39.54 kJ L^−1^ (8).

#### Carbon and Nitrogen Balance

In the C-N method, it is assumed that all energy is retained in the form of fat or protein (9), and the RE is determined based on the analysis of the C-N balance. C balance is the total amount of C retained in the body, and the amount of C retained in fat can be calculated by subtracting the amount of C retained in protein determined by N balance from the C balance. Assuming that fat has an energy equivalent of 39.76 kJ g^−1^ and contains 0.767 C and protein has an energy equivalent of 23.86 kJ g^−1^ and contains 0.16 N and 0.52 C, the RE in fat (REfat) and protein (REprotein) can be calculated. Energy retained as fat (REf) and protein (REp) can be calculated as follows:

- REp = N balance (g) × 6.25 × 23.86;
- REf = (C balance (g) - N balance (g) × 6.25 × 0.52) × 1.304 × 39.76 (2).
- RE can be calculated as RE = REp + REf according to Brouwer (8).

#### Requirements of Energy and Protein for Maintenance

The difference between the metabolic energy intake (MEI) and the retained energy was thought to be heat production (HP). According to the method described by Lofgreen and Garrett (10), the antilog of the linear regression intercept between the HP and MEI logarithms was used to estimate the net energy demand for maintenance (NEm, kJ kg^−1^ BW^0.75^) of male sika deer. According to the method proposed by Galvani et al. (11), the ME requirement for maintenance (MEm, kJ kg^−1^BW^0.75^) was calculated by iterating the semi-logarithmic linear regression equation until HP was equal to MEI. The maintenance efficiency (km) of ME was calculated as NEm/MEm.

The net protein requirement for maintenance was estimated by a linear regression equation between the daily retained N (RN; mg kg^−1^ BW^0.75^) and the daily N intake (NI, g kg^−1^ BW^0.75^). The intercept of this regression equation represents the loss of endogenous and metabolic N. The result of multiplying the loss by factor 6.25 was assumed to be the net protein requirement for maintenance (NPm, g kg^−1^ BW^0.75^).

### Statistical Analysis

Data were presented as means ± SD. The effects of feeding levels on the apparent digestibility of nutrients, energy values, energy balance, carbon-nitrogen balance, CH_4_, and CO_2_ emissions were analyzed using PROC GLM of SAS 8.0 (SAS Institute, Inc.; Cary, NC). The differences among the treatments were considered statistically significant with *P* < 0.05.

## Results

### DM Intake and Energy Balance

The effects of feed intake on BW, dry matter intake (DMI), and energy balance in the digestion and respirometry trials are present in Table 2. Different feeding levels had a significant impact on DM intake (*P* < 0.05). The feed levels significantly affected the BW of deer. The BW decreased significantly with the decrease of feed levels. As feed intake increased, total energy intake (GEI), fecal energy (FE), urine energy (UE), DE, ME, and methane energy significantly decreased (*P* < 0.05), while DE/GE, methane energy/GE significantly increased (*P* < 0.05). There was no significant difference between ME/GE and ME/DE when the deer experienced different levels of diets (*P* > 0.05).

**Table 2:** Effect of feed intake on body weight, dry matter intake (DMI), and energy balance in the digestion and respirometry tiral.

**Item^a^**	**Feed level**				***P*-value**
	**100%**	**80%**	**60%**	**40%**	
DMI (kg d^−1^)	1.90 ± 0.12^a^	1.72 ± 0.25^b^	1.14 ± 0.13^c^	0.76 ± 0.06^d^	<0.001
BW^1^ (kg)	64.26 ± 3.87	62.15 ± 4.36	59.83 ± 3.83	56.43 ± 4.17	<0.001
GE^1^ intake (kJ kg^−1^BW^0.75^d^−1^)	1174.51 ± 109.56^a^	1080.20 ± 103.21^b^	743.48 ± 78.31^c^	517.89 ± 57.62^d^	<0.001
Fecal energy (FE) (kJ kg^−1^BW^0.75^d^−1^)	385.24 ± 15.87^a^	339.32 ± 21.56^b^	206.09 ± 20.31^c^	118.79 ± 12.57^d^	<0.001
Urinary energ y (UE) (kJ kg^−1^BW^0.75^d^−1^)	36.40 ± 3.51^a^	34.01 ± 2.87^b^	23.35 ± 3.56^c^	16.87 ± 2.91^d^	<0.001
Methane energy (kJ kg^−1^ BW^0.75^d^−1^)	76.34 ± 7.31^a^	73.04 ± 8.52^a^	55.91 ± 7.63^b^	43.01 ± 5.45^c^	0.035
Methane energy/GE (%)	6.48 ± 1.13^a^	6.69 ± 0.89^a^	7.52 ± 1.25^b^	8.32 ± 1.37^c^	0.631
DE^1^ (kJ kg^−1^ BW^0.75^d^−1^)	769.27 ± 20.51	745.88 ± 25.87	537.39 ± 24.01	399.10 ± 18.75	<0.001
MEI^1^ (kJ kg^−1^ BW^0.75^d^−1^)	752.87 ± 35.05^a^	686.87 ± 28.67^a^	434.04 ± 20.55^b^	286.23 ± 18.96^c^	<0.001
DE/GE (%)	65.50 ± 7.82^a^	68.98 ± 8.31^a^	72.27 ± 7.69^b^	77.18 ± 7.53^c^	0.023
ME/GE (%)	64.10 ± 6.55	65.46 ± 7.69	69.18 ± 5.81	74.27 ± 7.13	0.045
ME/DE (%)	95.38 ± 9.66	95.29 ± 8.75	94.89 ± 8.94	94.44 ± 9.02	0.532

*^1^BW, body weight; GE, gross energy; DE, digestible energy; ME, metabolisable energy; MEI, ME intake.*

a−d *In the same row, values without a different superscript differ (P < 0.05)*.

### CH_4_ and CO_2_ Emissions

The effects of feed intake level on CH_4_ and CO_2_ emissions are shown in Table 3. Feed intake level significantly affected CH_4_ emission. CH_4_ emission (L d^−1^; L kg^−1^ BW^0.75^ d^−1^) decreased (*P* < 0.05) as the feeding level decreased in the growing period. CH_4_ emission (L kg^−1^ DMI) showed an opposite trend (*P* < 0.05).

**Table 3:** Effect of feed intake on daily methane (CH_4_) and carbon dioxide (CO_2_) emissions.

**Item^**1**^**	**Feed level**				***P*-value**
	**100%**	**80%**	**60%**	**40%**	
**CH**_**4**_ **emission**
L d^−1^	61.26 ± 6.13^a^	50.15 ± 6.03^b^	41.82 ± 3.97^c^	30.53 ± 3.66^d^	0.012
L kg^−1^ BW^0.75^d^−1^	2.70 ± 0.38^a^	2.27 ± 0.42^a^	1.89 ± 0.35^b^	1.77 ± 0.31^c^	0.021
L kg^−1^DMI	29.24 ± 3.59^a^	31.16 ± 3.15^a^	35.81 ± 3.52^b^	48.07 ± 5.03^c^	0.035
**CO**_**2**_ **emission**
L d^−1^	610.50 ± 50.21^a^	530.25 ± 48.33^b^	480.36 ± 36.53^c^	389.55 ± 36.21^d^	<0.001
L kg^−1^ BW^0.75^d^−1^	26.92 ± 3.27^a^	23.94 ± 2.46^b^	22.33 ± 2.08^b^	18.93 ± 1.88^c^	0.038
L kg^−1^DMI	308.32 ± 29.56^a^	321.28 ± 28.57^b^	430.06 ± 35.41^c^	512.57 ± 49.32^d^	<0.001

*^1^BW, body weight; DMI, dry matter intake*.

a−d *In the same row, values without a different superscript differ (P < 0.05)*.

At the same time, feed intake level also produced significant effects on CO_2_ emission. The CO_2_ emission (L d^−1^; L kg^−1^ BW^0.75^ d^−1^) decreased (*P* < 0.05) as the feeding level decreased. However, CO_2_ emission (L kg^−1^ DMI) showed an opposite trend (*P* < 0.01).

### C-N Balance

Table 4 shows the effects of feed intake level on daily C-N balance, retained energy, and heat production, fecal C, urinary, and retention C were significantly affected by the level of feed intake (*P* < 0.05). The above indicators showed a significant downward trend. The content of CO_2_-C and CH_4_-C significantly decreased (*P* < 0.05) as feed intake level decreased, but the apparent digestibility of C (digestible C) was not affected by the feed intake level (*P* > 0.05).

**Table 4:** Effect of feed intake on carbon (C) and nitrogen (N) balances (g kg^−1^ BW^0.75^ d^−1^), heat production (HP) (kJ kg^−1^ BW^0.75^ d^−1^), and retained energy (RE) (kJkg^−1^ BW^0.75^ d^−1^) by sika deer.

**Item^**1**^**	**Feed level**				***P*-value**
	**100%**	**80%**	**60%**	**40%**	
**C (g kg** ^**−1**^ **BW** ^**0.75**^ **d** ^**−1**^ **)**					
Intake	50.52 ± 6.03^a^	42.69 ± 4.31^b^	35.21 ± 5.38^c^	24.75 ± 3.51^d^	<0.001
Fecal	24.22 ± 3.56^a^	19.51 ± 2.43^b^	14.34 ± 2.65^c^	9.13 ± 2.41^d^	0.011
Urinary	1.82 ± 0.09^a^	1.53 ± 0.21^b^	1.21 ± 0.18^c^	0.96 ± 0.25^d^	0.013
CO_2_-C	14.67 ± 2.56^a^	13.05 ± 1.08^a, b^	12.17 ± 2.56^b^	10.32 ± 2.37^c^	0.032
CH4-C	1.56 ± 0.23^a^	1.31 ± 0.32^a^, ^b^	1.09 ± 0.33^b^	1.02 ± 0.19^c^	0.021
Retention C	8.25 ± 0.35^a^	7.27 ± 0.56^a^, ^b^	4.40 ± 0.57^b^	2.32 ± 0.27^c^	
Apparent C digestibility (%)	52.06 ± 4.82	54.30 ± 4.32	59.27 ± 6.03	63.11 ± 7.38	0.2232
**N (g kg** ^**−1**^ **BW** ^**0.75**^ **d** ^**−1**^ **)**					
Intake (NI)	2.12 ± 0.35^a^	1.96 ± 0.26^b^	1.34 ± 0.31^c^	0.93 ± 0.17^d^	0.018
Fecal (FN)	1.10 ± 0.12^a^	0.98 ± 0.10^b^	0.54 ± 0.11^c^	0.32 ± 0.05^d^	0.022
Urinary (UN)	0.63 ± 0.11^a^	0.61 ± 0.09^a^, ^b^	0.41 ± 0.08^b^	0.30 ± 0.04^c^	0.017
Retention N(RN)	0.38 ± 0.08^a^	0.30 ± 0.04^a^, ^b^	0.24 ± 0.05^b^	0.20 ± 0.02^c^	0.020
Apparent N digestibility (%)	48.04 ± 4.13	50.10 ± 4.87	59.70 ± 5.61	65.72 ± 5.88	0.235
Protein deposited (g kg^−1^ BW^0.75^d^−1^)	2.38 ± 0.21^a^	1.88 ± 0.36^b^	1.50 ± 0.32^c^	1.38 ± 0.22^d^	0.031
Rep (kJ kg^−1^ BW^0.75^d^−1^)	56.66 ± 4.87^a^	44.74 ± 3.96^b^	35.79 ± 3.96^c^	32.81 ± 4.57^d^	0.043
REf (kJ kg^−1^ BW^0.75^d^−1^)	363.71 ± 30.52^a^	326.38 ± 29.56^b^	187.69 ± 25.31^c^	83.21 ± 9.34^d^	0.012
RE (kJ kg^−1^ BW^0.75^d^−1^)	420.31 ± 40.31^a^	371.08 ± 32.31^b^	223.48 ± 30.67^c^	116.02 ± 13.52^d^	0.018
HP (kJ kg^−1^ BW^0.75^d^−1^)	332.50 ± 27.86^a^	315.76 ± 28.78^a^, ^b^	210.56 ± 25.36^b^	170.21 ± 19.34^c^	0.013

*^1^HP, heat production; RE, retained energy; REp, RE for protein; REf, RE for fat; NI, nitrogen intake; FN, fecal nitrogen;UN, urinary nitrogen; RN, retained nitrogen*.

a−d *In the same row, values without a different superscript differ (P < 0.05)*.

RE and HP significantly decreased with a decrease in feed intake (*P* < 0.05), so did the energy retention components REp and REf. NI, FN, UN, RN, and protein deposited significantly decreased (*P* < 0.05) as feed intake level decreased, but the apparent digestibility of N had no difference at different feeding levels (*P* > 0.05).

### Nutrient Apparent Digestibility

The effects of feed intake level on the apparent digestibility of dry matter (DM), organic matter (OM), acid detergent fiber (ADF), and neutral detergent fiber (NDF) are shown in Table 5. As the feed intake decreased, the digestibility of DM, OM, ADF, and NDF significantly increased (*P* < 0.05).

**Table 5:** Effect of feed intake level on DM, ADF and NDF intake, excretion and apparent digestibility by sika deer.

**Item**	**Feed level**				***P-*value**
	**100%**	**80%**	**60%**	**40%**	
**DM (g kg** ^**−1**^ **BW** ^**0.75**^ **d** ^**−1**^ **)**
Intake	83.74 ± 9.78^a^	77.69 ± 8.46^b^	53.00 ± 6.21^c^	36.93 ± 3.58^d^	<0.001
Fecal	41.69 ± 5.65^a^	35.88 ± 4.52^a^^b^	22.46 ± 3.51^b^	14.75 ± 2.42^c^	<0.001
Apparent DM digestibility (%)	50.21 ± 6.03^a^	53.82 ± 6.37^a^^b^	57.63 ± 6.84^b^	60.05 ± 6.82^c^	0.035
**OM (g kg** ^**−1**^ **BW** ^**0.75**^ **d** ^**−1**^ **)**					
Intake	71.18^a^	66.03^a^	45.05^b^	31.39^c^	0.038
Fecal	33.54^a^	30.17^a^	18.19^b^	12.11^c^	0.042
Apparent OM digestibility (%)	52.88^a^	55.31^a^	59.63b^c^	61.4^2^	0.022
**ADF (g kg** ^**−1**^ **BW** ^**0.75**^ **d** ^**−1**^ **)**					
Intake	13.53 ± 2.58^a^	12.55 ± 2.41^a^	8.56 ± 1.34^b^	5.97 ± 1.06^c^	<0.001
Fecal	9.45 ± 2.41^a^	8.46 ± 1.85^b^	5.52 ± 1.10^c^	3.58 ± 0.96^d^	<0.001
Apparent ADF digestibility (%)	30.12 ± 5.61^a^	32.56 ± 4.23^b^	35.43 ± 4.36^bc^	40.05 ± 4.31^c^	0.033
**NDF (g kg** ^**−1**^ **BW** ^**0.75**^ **d** ^**−1**^ **)**					
Intake	34.78 ± 2.56^a^	32.27 ± 3.52^a^^b^	22.02 ± 3.15^b^	15.34 ± 2.06^c^	<0.01
Fecal	24.51 ± 3.87^a^	21.55 ± 2.69^b^	13.89 ± 2.06^c^	8.99 ± 1.87^d^	<0.001
Apparent NDF digestibility (%)	29.53 ± 2.45^a^	33.21 ± 4.57^b^	36.91 ± 4.05^bc^	41.37 ± 5.04^c^	0.021

*^1^BW, body weight; DM, dry matter; OM, organic matter; ADF, acid detergent fiber; NDF, neutral detergent fiber*.

a−d *In the same row, values without a different superscript differ (P < 0.05)*.

### Requirements of Energy and Net Protein for Maintenance

**Figure 1:**
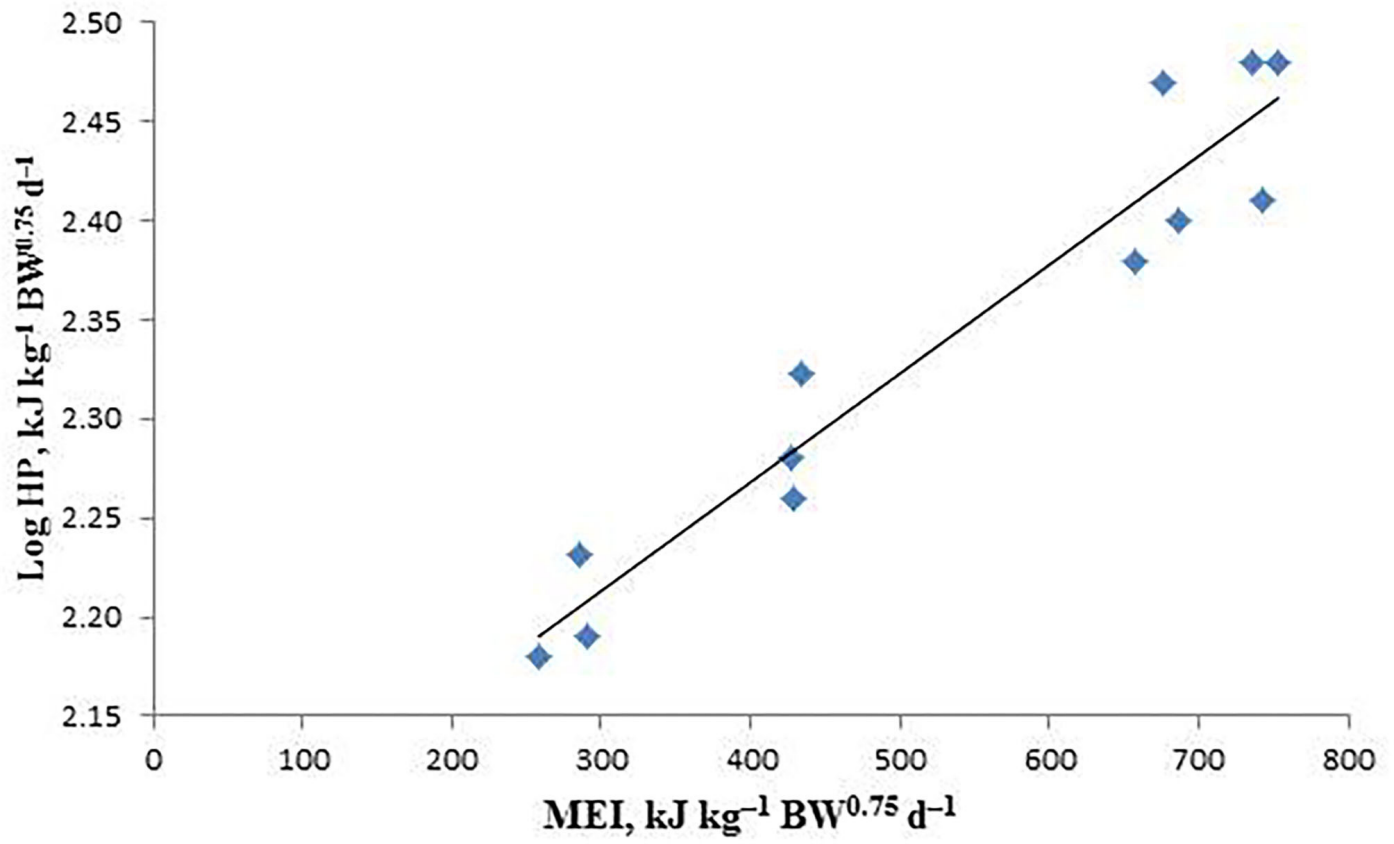
The relationship between the logarithm of heat production (HP) and metabolizable energy intake (MEI) of sika deer in the indirect calorimetry method. LogHP = 2.3495(± 0.0212) + 0.0005(± 0.0002) × MEI; *R*^2^ = 0.9246, *P* < 0.001, *n* = 12.

The estimated values of MEm, NEm, and NEm/MEm (Km) are shown in Table 6, and the linear relationship between logHP and MEI is also shown in Figure 1. The NEm value of the male sika deer was determined to be 223.62 kJ kg^−1^ BW^0.75^d^−1^ by calculating the antilog of the regression intercept, and the MEm value was calculated to be 251.17 kJ kg^−1^ BW^0.75^d^−1^ through iteration of the regression equation between Log HP on MEI until HP is equal to MEI. Meanwhile, the Km (NEm/MEm) value was calculated to be 0.89.

**Table 6:** Estimates of heat production (HP, kJ kg^−1^BW^0.75^d^−1^) and metabolisable energy (ME) intake (MEI, kJ kg^−1^BW^0.75^d^−1^) in the equation to predict net energy requirement for maintenance of sika deer.

**BW, kg**	**Equation**	**RMSE^a^**	***R* ^**2**^**	**Number of deer**	***P*-value**	**NEm (kJ kg^**−1**^ BW^**0.75**^d^**−1**^)**	**MEm (kJ kg^**−1**^ BW^**0.75**^ d^**−1**^)**	**Km**
56.43–64.26	LogHP = 2.3495(± 0.0212) + 0.0005(± 0.0002) × MEI	0.0831	0.9246	12	<0.001	223.62	251.17	0.89

a *RMSE, root mean square error; Km, the efficiency of ME utilization for maintenance was computed as NEm/Mem*.

**Figure 2:**
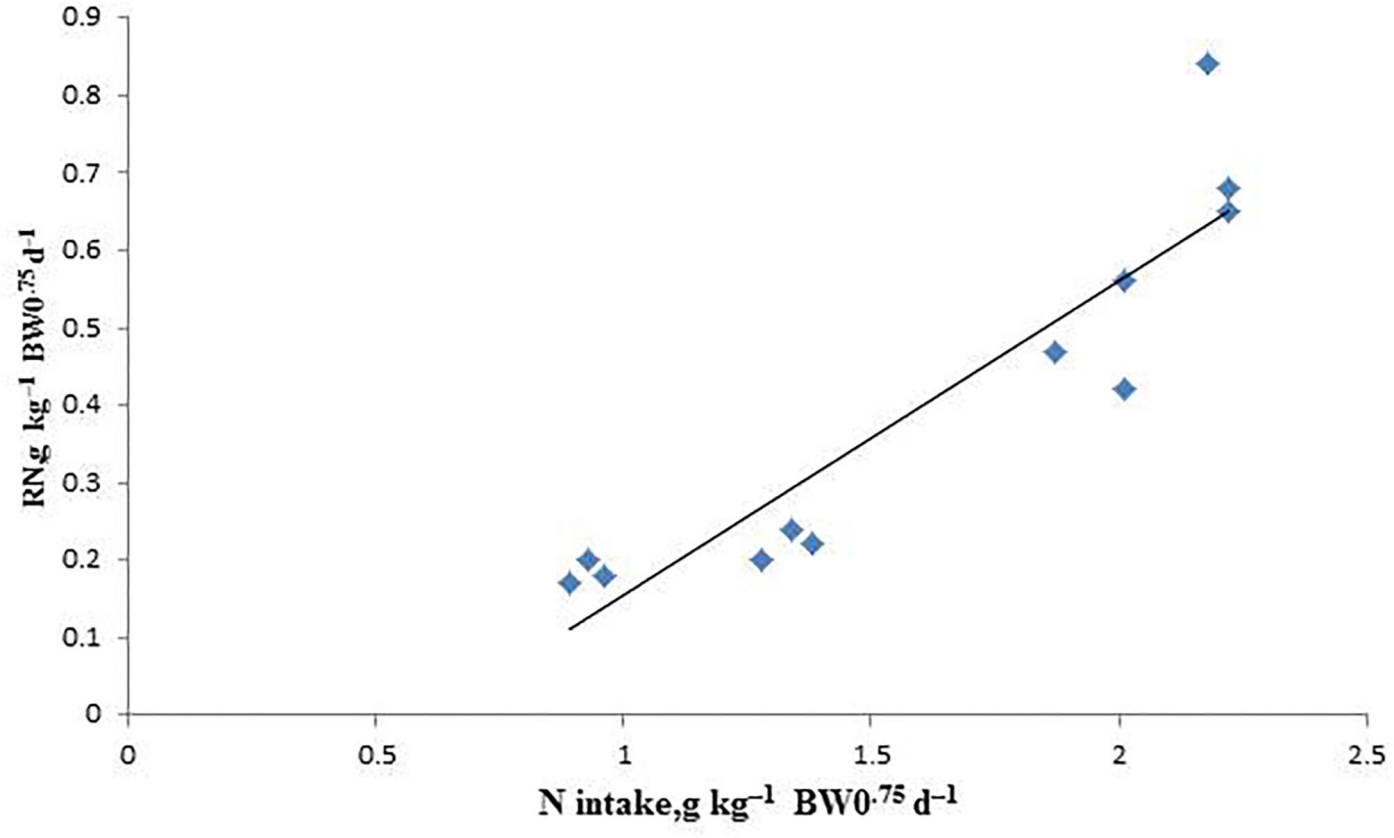
The relationship between the retained nitrogen (RN) and nitrogen intake (NI) of arctic foxes in the indirect calorimetry method. RN = −0.2518(± 0.1352) + 0.407(± 0.0524) × NI. *R*^2^ = 0.8479, *P* < 0.001, *n* = 12.

The linear relationship between RN and NI is shown in Table 7, Figure 2. Endogenous and metabolic loss of N was calculated as 251.8 mg kg^−1^ BW^0.75^d^−1^ by estimating the intercepts of the linear regression of RN on NI. The NPm value was calculated to be 2.045 g kg^−1^ BW^0.75^d^−1^ for growing male sika deer.

**Table 7:** Estimates of retained N (RN, g kg−1 BW0.75 d−1) and N intake (NI, g kg^−1^ BW^0.75^ d^−1^) in the equation to predict net protein requirement for maintenance (NPm, g kg^−1^ BW^0.75^ d^−1^).

**BW, kg**	**Equation**	**RMSE^a^**	***R* ^**2**^**	**Number of deer**	***P*-value**	**NNm (mg kg^**−1**^BW^**0.75**^ d^**−1**^)**	**NPm (g kg^**−1**^ BW0^**.75**^ d^**1**^)**
56.43–64.26	RN = −0.2518(± 0.1352) +0.407(± 0.0524) × NI	0.1235	0.8479	12	<0.001	251.8	1.57

a *RMSE, root mean square error; NNm, net N requirement for maintenance (mg kg^−1^ BW^0.75^ d^−1^) calculated as the intercept of this regression; NPm = NNm × 6.25; BW, body weight*.

## Discussion

### Energy Balance and C-N Balance

The CH_4_ emission rate is a key factor used to assess the potential degree of global warming and to estimate enteric CH_4_ estimation (12). The results of this study show that the CH_4_ emission rate increased as the feeding level decreased for male sika deer during their growing period. It may be an important strategy to increase feeding during the above maintenance period to reduce enteric CH_4_ emission (13), which is consistent with the results of this study. For Dorper crossbred ram lambs, CH_4_ energy/GE increased but DE/GE, ME/GE, and ME/DE decreased in their growing period as the feeding level increased (6). These are consistent with the results for growing male sika deer in this study, except for ME/DE, which was not affected by feed intake. In this study, the ME/DE of male sika deer was 95.01%, which was higher than that of lamb (6), broiler (14), and sheep (15). This may be related to different dietary components and interspecies differences.

Flatt (16) found that DE and CH_4_ energy of cows decreased but their urinary energy remained unchanged during their pregnancy. In contrast, Ferrell et al. (17) reported that any difference in energy digestibility of heifers due to pregnancy was insignificant compared to differences in feeding levels. The current data of male sika deer during their growing period suggest that the methane energy, urinary energy, fecal energy, and DE increased as the feed intake level increased. The results of this study are consistent with those reported above.

Blaxter (18) found that because the C-N balance method involved more analysis and measurement than the energy balance method, errors of about 30% may be encountered. As revealed by Kishan et al. (19), for male buffaloes and crossbred cattle, energy levels affected the excretion of C and N in urine, the C in urine was significantly related to DE intake (*P* < 0.01), but the urinary, CO_2_, and CH_4_ carbon outgo were not affected. There is also a correlation between N excretion and urinary C content (20). These are consistent with the results of this study. Furthermore, the results of this study show that the apparent digestibility of C was 52–63%, which is consistent with the results reported by Blaxter and Wainman (20). There is a certain difference between RC and RN calculated using the C-N balance method in this study. Graham (21) found that the RN calculated using the comparative slaughter method decreased as the number of gestation days increased. In this study, RN and RE decreased as the levels of feeding decrease. This is consistent with the results of Zhang et al. (15), Singh et al. (22), and George et al. (23).

Ferrell (24) found that energy intake affected HP because of the metabolic activity of visceral organs. As the MEI of growing cattle increased, HP increased exponentially (25–27). As indicated by the results of Taylor and Turner (28), HP increased as the nutrient level increased, which is mainly due to the increased metabolism associated with energy retention. Analysis of energy metabolism for growing cattle indicated that HP increased exponentially with an increase in MEI level (25). It has been well-established that HP will increase significantly during pregnancy (29). In this study, MEI and HP gradually decreased, accompanied by a decrease in feeding levels, which may be due to the thermal effect of reduced feed. Meanwhile, ME intake decreased, leading to a decrease in HP (26, 30, 31). These are consistent with the findings in growing and fattening pigs reported by Zhang et al. (32).

The REp value is much lower than REf value. When deer were fed at 40% of the intake level, REp and REf reached their minimum values. These results are consistent with the findings in Hu sheep (15) and in arctic foxes (33). In this study, the decrease in the feed intake of male sika deer led to the reduction of NI, FN, and UN. As the feed intake decreased, RN gradually decreased from 0.38 to 0.20 g kg^−1^ BW^0.75^d^−1^. This is consistent with the findings of Singh et al. (22).

### Nutrient Digestibility

The digestibility of nutrients in the rumen is the competition result between digestion and passing rate. Among them, the passing rate is positively correlated with dry matter intake (34). Degen and Young (35) found a correlation between increased digesta passage rates and increased feed intake. In this study, the digestibility values of C, N, DM, OM, ADF, and NDF increased significantly with a decrease in diet intake, indicating that dietary restriction can improve the digestibility and utilization of nutrients. The deer body promotes nutrient digestibility and utilization to meet maintenance requirements while feed intake is less. Deng et al. (6) found that the apparent energy digestibility (DE/GE) and metabolic rate (ME/GE) of lambs fed *ad libitum* were lower than lambs fed in a restricted manner. The results on male sika deer in this study are consistent with the above research results.

### Energy and Protein Requirements for Maintenance

The logarithmic relationship between MEI and HP is often used to calculate NEm. HP is equal to NEm in the case of zero MEI (10, 26). Similarly, MEm can also be calculated by extrapolating HP where it is equal to MEI. The values of NEm and MEm calculated by the regression equation were 223.62 and 251.17 kJ kg^−1^ BW^0.75^d^−1^, respectively. The Km was calculated to be 0.89 in this study. Li et al. (36) studied the energy metabolism of adult male sika deer and determined that the requirement of MEm of adult sika deer was 516 kJ kg^−1^ BW^0.75^d^−1^. The result is greater than that in this study. The reason may be the different ages of the deer selected for experiments and the different physiological states of each period. Adult deer need more energy to maintain their growth and antler. In contrast, for deer in the growing period, more energy is used for the development and growth of their own bodies, and thus less MEm was observed in them. The Km value in this study is higher than that (0.707) reported by Li (36) in adult deer and that of lambs and sheep (14, 15, 32). This may also be related to the age of the selected deer. Luo et al. (37) reported that the difference in MEm seems to depend mainly on the change in NEm rather than Km, which can explain the difference in MEm requirements. The values of NEm and MEm vary from species to species. For animals, the species, physiological stages, environmental temperature, and feed composition also affect the values of NEm and Mem (14, 38). MEm was 768 kJ kg^−1^ BW^0.75^d^−1^ and 501 kJ kg^−1^ BW^0.75^d^−1^ at 18 and 24°C, respectively for adult female mink (38). In this study, male sika deer with a bodyweight of 56.43–64.26 kg were selected. Meanwhile, the temperature in the respiration chamber was 22°C, and the male sika deer in Northeast China during their growing period was chosen. These may be the main reasons why the NEm and MEm values are different from those in previous reports (36).

ARC (39) assumed that NPm equal to the amount of protein that can offset the loss of urine, feces, and dermal N, except for growing lambs because they do not consider dermal loss ARC. For lambs and sheep (15, 40), the regression equation between RN and daily NI is an effective method to obtain NPm through N-balance trails. The intercept of the regression equation represents the endogenous and metabolic N loss. According to the regression equation between the daily NI and RN of the male sika deer during their growing period, the values of NNm and NPm were estimated to be 251.8 mg kg^−1^ BW^0.75^d^−1^ and 2.045 g kg^−1^BW^0.75^d^−1^, respectively. Chizzotti et al. (26) reported that the NPm estimated according to the relationship between RN and daily NI using the comparative slaughter method is greater than the NPm determined based on the relationship between RN and daily NI using the N-balance method. This discrepancy may be due to losses of N that are not accounted for by the N balance method. The scurf protein represents about 20% of the maintenance requirement of the ARC system. However, no data was present in growing male sika deer. The N balance trails can reduce animal injuries and meet animal welfare requirements and is an important method for estimating net protein requirement.

## Conclusions

In conclusion, the MEm and NEm values of growing male sika deer were estimated to be 223.62 and 251.17 kJ kg^−1^ BW^0.75^d^−1^, respectively, according to the logarithmic regression between the HP and MEI The NNm and NPm values of growing male sika deer were estimated to be 251.8 mg and 2.045 g kg^−1^BW^0.75^d^−1^, respectively, based on the linear regression relationship between daily NI and RN. These results fill the gap in the research on the net energy and protein requirements of male sika deer and provide basic data for determining the nutritional requirements of sika deer in China.

## Data Availability Statement

The original contributions presented in the study are included in the article/supplementary material, further inquiries can be directed to the corresponding author/s.

## Ethics Statement

The animal study was reviewed and approved by Animal Care and Use Guidelines of the Institute of Special Animal and Plant Science.

## Author Contributions

KB: conceived the study, managed the animals, and oversaw the statistical analysis and manuscript preparation. XW: sample collection. KW: statistical analysis and drafted the manuscript. GL: statistical analysis and manuscript preparation. HL: manuscript preparation. All authors contributed to the article and approved the submitted version.

## Funding

This study was funded by the National Key R&D Program of China (2018YFC1706602), the National Natural Science Foundation of China (20170101034JC), the science and technology project (20190304007YY) of Jilin Province, and Science and Technology Innovation Project of the Chinese Academy of Agricultural Sciences (number CCCS-ASTIP-2016-IS APS).

## Conflict of Interest

The authors declare that the research was conducted in the absence of any commercial or financial relationships that could be construed as a potential conflict of interest.

## Publisher's Note

All claims expressed in this article are solely those of the authors and do not necessarily represent those of their affiliated organizations, or those of the publisher, the editors and the reviewers. Any product that may be evaluated in this article, or claim that may be made by its manufacturer, is not guaranteed or endorsed by the publisher.
